# MiRNAs prognostic for basal and BRCA1 breast cancer

**DOI:** 10.18632/oncotarget.26297

**Published:** 2018-11-06

**Authors:** Bhupinder Pal, Robin L. Anderson

**Affiliations:** Bhupinder Pal: Translational Breast Cancer Program, Olivia Newton-John Cancer Research Institute, Heidelberg, Australia; School of Cancer Medicine, La Trobe University, Bundoora, Australia

**Keywords:** BRCA1, miRNA, basal breast cancer

Since the first description of microRNAs in *C. elegans* in 1993, there has been an explosion in knowledge of the number, sequence and function of microRNAs that regulate all systems and pathways in cells and organisms across the plant and animal kingdoms. Their primary role is to regulate protein translation and many studies have now revealed their oncogenic or tumour suppressor functions in cancer. In the cancer setting, the expression of miRNAs is often deregulated due to aberrant transcriptional changes resulting from point mutations, chromosomal alterations, epigenetic changes or aberrant miRNA biogenesis [1]. Alongside the functional studies, an increasing number of reports have revealed their value as diagnostic or prognostic biomarkers in various cancers, including breast [2]. Large studies have associated deregulated miRNA expression with tumour classification, diagnosis and prognosis across multiple cancers [2].

In breast cancer, loss of the tumour suppressor gene, breast cancer 1 (*BRCA1*), is usually associated with aggressive basal-like hormone receptor negative tumours, although a minority are of the ER positive subtype. BRCA1 is involved in diverse cellular functions including DNA damage-induced cell cycle checkpoint activation, DNA damage repair, protein ubiquitination, chromatin remodelling, apoptosis as well as transcriptional regulation of coding and non-coding regions [3]. An intricate relationship between BRCA1 and the microRNA network is gradually emerging. It is now known that the *BRCA1* transcript is not only a target of post-transcriptional regulation by a number of miRNAs, but also directly or indirectly is involved in transcriptional regulation of other microRNAs [4]. For some microRNAs, BRCA1 is involved in the processing of precursor-miRs via the DROSHA complex and Smad3/p53/DHX9 [5].

In a recent study, Milevskiy and colleagues used *Brca1* null mice to identify 140 differentially expressed miRNAs, nine of which were also found to be deregulated in mutant BRCA1 breast tumours [6]. Of these, miR 34b-5p, miR-744-5p, miR-485-3p and miR-542-3p were upregulated, whereas miR-664-3p, miR-221-3p, miR-16-5p, miR-29b-1-5p and miR-30b-5p were down in the *Brca1* null mouse mammary gland and down in *BRCA1* breast tumours. To identify the underlying molecular mechanism responsible for miRNA deregulation due to *BRCA1* loss, the authors utilised published ChIPseq data from human breast epithelial tissue using a BRCA1 antibody. The ChiPseq profile identified putative BRCA1 binding sites present 15kb upstream of promoter regions of seven of the above nine differentially expressed miRNAs. To validate these bioinformatic observations, the authors expressed the wild type *BRCA1* gene in the HCC1937 cell line that lacks functional BRCA1 protein. Interestingly, this approach had no significant transcriptional impact on six of these seven miRNAs, rescuing expression only of miR-29b-1-5p. These results indicate that BRCA1 alone is not sufficient to alter expression of the other miRNAs that possibly require co-factors absent in HCC1937 cells.

Based on their observations that wild type BRCA1 binds to putative cis-elements present 15kb upstream in the promoter region of miR-29b-1-5p and increases its expression, the authors were prompted to assess the prognostic value of miR-29b-1-5p by the multivariate cox-proportional model and by Kaplan-Meier analysis using the METABRIC and TCGA breast cancer cohorts. Remarkably, the univariate and multivariate analyses revealed that high miR-29b-1-5p expression was strongly associated with improved overall survival in breast cancer patients with basal or hormone receptor negative (TNBCs) tumours. In fact miR-29b-1-5p stratified overall survival better than standard markers such as lymph node involvement or tumour size or grade. Interestingly, miR-664 expression, which did not respond to *BRCA1* induction in HCC1937 cell line, was also strongly associated with the overall survival of patients with TNBC and basal-like tumours. MiR-664b-5p was shown recently to increase chemosensitivity to PARP inhibitors by targeting oncogenic Cyclin E2 (CCNE2) in BRCA1 deficient HCC1937 cells [7].

Other global miRNA expression studies in breast cancer have also reported downregulation of the miR-29 family in a subset of BRCA-X, BRCA1 and BRCA2 breast tumours [8, 9], which suggests that the transcription of the miR-29 family is regulated by a complex transcriptional mechanism in presence or absence of BRCA1. Therefore, the identification of BRCA1-cis element mediated miRNA expression has wider implications, but this mechanism needs to be further validated in other cancer cell lines, preferably using cis-element mediated reporter assays.

**Figure 1:**
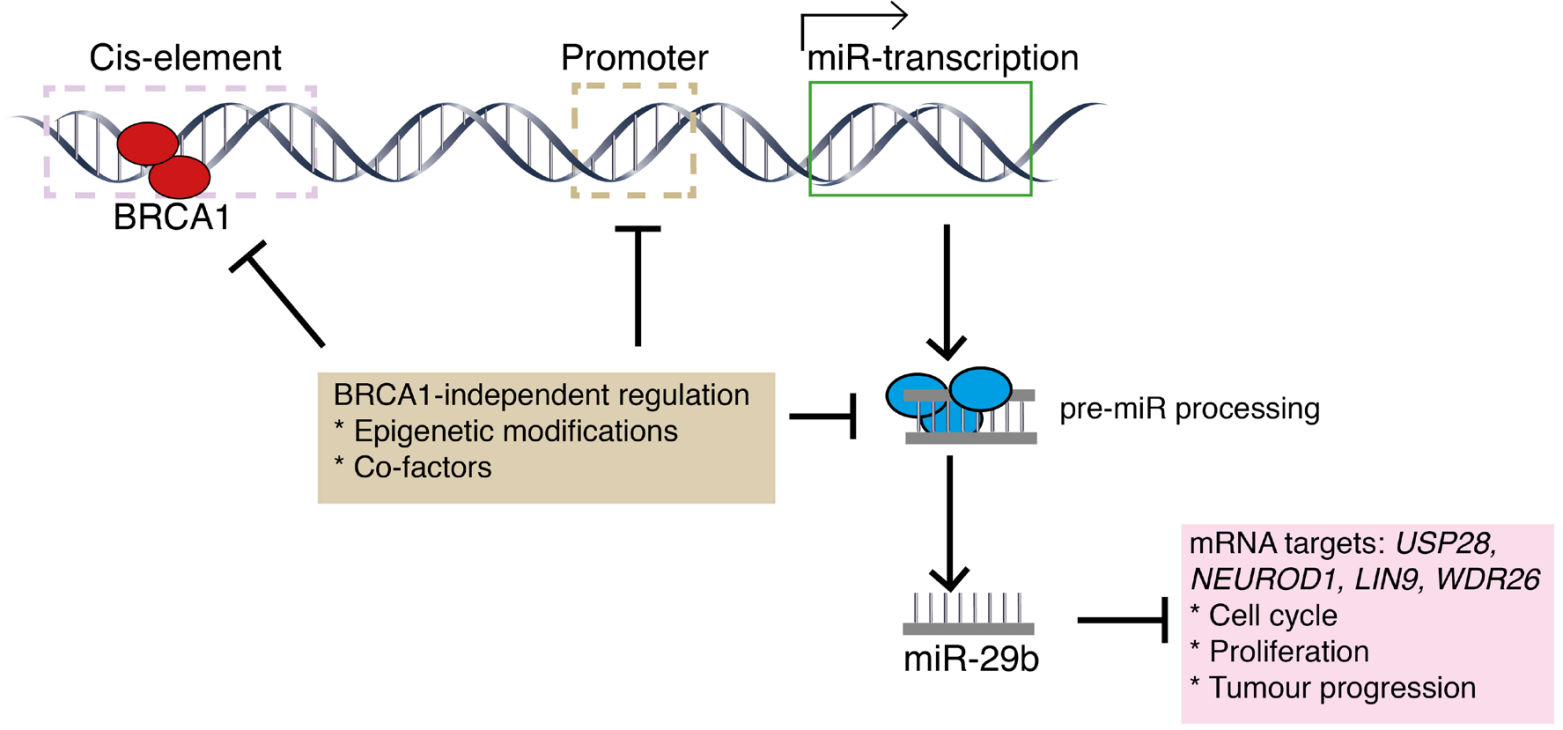
Schematic representation of miR-29b biogenesis BRCA1 binds to putative cis-elements present upstream of miR29b to mediate transcriptional induction. In TNBC/basal tumours, possible reasons for miR-29b downregulation are the binding of unknown repressive cofactors at cis-elements, or epigenetic modifications and/or negative regulation of miRNA-DROSHA processing machinery.

Milevskiy et al have identified a novel non-canonical function of BRCA1 involving transcriptional upregulation of miR-29b-1-5p, which may be required for its tumour suppressor activity and maintenance of genomic stability (Figure 1). The authors used a published algorithm to predict protein targets of miR-29b-1-5p, finding that the top candidates, USP28, NEUROD1, LIN9 and WDR26 have been associated with breast cancer. Now, the question remains as to its functional relevance in breast cancer progression and whether any of these target proteins are involved and/or could become therapeutic targets. Further, the mechanism responsible for the transcriptional down-regulation of miR-29b-1-5p in non-BRCA1 basal tumours remains to be resolved.
